# The Reappraisal of the Reappraisal—CRAC Channels Are Activated by L-Type Ca^2+^ Channel Blockers, Reply to Bird et al

**DOI:** 10.1093/function/zqae007

**Published:** 2024-02-02

**Authors:** Mohamed Trebak, Khaled Machaca, Patrick G Hogan

**Affiliations:** Department of Pharmacology and Chemical Biology, University of Pittsburgh School of Medicine, Pittsburgh, PA 15213, USA; Vascular Medicine Institute, University of Pittsburgh, Pittsburgh, PA 15213, USA; UPMC Hillman Cancer Center, University of Pittsburgh, Pittsburgh, PA 15213, USA; Calcium Signaling Group, Research Department, Weill Cornell Medicine Qatar, Education City, Qatar Foundation, Doha, 00000, Qatar; Department of Physiology and Biophysics, Weill Cornell Medicine, New York, NY 10075, USA; La Jolla Institute for Immunology, La Jolla, CA 92037, USA; Moores Cancer Center, University of California San Diego, La Jolla, CA 92037, USA; Program in Immunology, University of California San Diego, La Jolla, CA 92037, USA

## A Perspective on “A Reappraisal of the Effects of L-Type Ca^2+^ Channel Blockers on Store-Operated Ca^2+^ Entry and Heart Failure”

Over a decade ago, we showed that differentiated, contractile arterial myocytes do not express functional Ca^2+^ release-activated Ca^2+^ (CRAC) channels that mediate store-operated Ca^2+^ entry (SOCE). However, CRAC currents emerge in dedifferentiated fibroproliferative noncontractile arterial myocytes because of increased expression of Stromal Interacting Molecule (STIM1) and Orai1.^1,2^ Dedifferentiated myocytes (called synthetic) are a hallmark of neointimal hyperplasia in diseases such as hypertension, restenosis, and atherosclerosis. The emergence of CRAC channels is only one aspect of synthetic myocytes. Synthetic myocytes remodel their ion channel repertoire to promote hyperplasia and migration at the expense of excitability and contractility. Synthetic myocytes downregulate the major orchestrators of excitation-contraction coupling: dihydropyridine-sensitive voltage-gated L-type Ca^2+^ channels (Ca_v_1.2) and ryanodine receptors (for review^3^).

Recently, we examined the effects of Ca_v_1.2 channel antagonists on synthetic arterial myocytes.^4^ We reported that Ca_v_1.2 channels blockers at concentrations ranging from 0.5 to 20 µm increased intracellular Ca^2+^ in synthetic arterial myocytes. Our data support the idea that Ca_v_1.2 channel blockers, including amlodipine, nifedipine, verapamil, and diltiazem activate STIM proteins and CRAC channels independently of store depletion.^4^We further showed that 0.5 µm amlodipine synergizes with suboptimal concentrations of the platelet-derived growth factor (0.5 ng/mL PDGF) in a STIM1-dependent manner to induce arterial myocyte proliferation and migration to levels approaching those produced by maximal PDGF stimulation (10 ng/mL). Bird et al.^5^ challenged our study and contended that our findings represent an artifact of fluorescence Ca^2+^ measurements because “amlodipine is strongly fluorescent in the cytoplasm with an excitation spectrum that overlaps with that of fura-2.” They reported Ca^2+^ imaging experiments using the single-excitation/single-emission dye Cal520 but did not attempt to replicate our key biological observations, most notably the synergistic effects of 0.5  μm amlodipine on PDGF-triggered arterial myocyte proliferation and migration, and their dependence on STIM1 (Figure 1H-K; Figure 7A-G; Figure S8).^4^ We reject the contention of Bird et al. that our conclusions are incorrect due to an unrecognized contribution of amlodipine fluorescence to the Ca^2+^ signal, based on the following evidence:

1. Contrary to the assertion of Bird et al., we have recognized the issue of extracellular amlodipine fluorescence, which we acknowledged, discussed, and corrected for in our Johnson et al. paper (first paragraph in “Results” and Supplemental Material, *Single Cell Ca^2+^ Imaging*).^4^ This was also recognized in our previous Liu et al. paper (“Methods,” *Ca^2+^ measurements*).^6^ Amlodipine did not create a false fura-2 fluorescence-ratio signal that would be misinterpreted as a Ca^2+^ increase. If it did, that signal would have been apparent in Figure 2I and J where amlodipine was added to STIM1/STIM2 double knockout cells or Orai1/Orai2/Orai3 triple knockout cells, or in Figure 4A and B where amlodipine was applied to cells in 0 m m Ca^2+^ extracellular solution, or in Figure 6K and L where amlodipine was applied to STIM1/STIM2 double knockout cells expressing STIM1 with N-terminal deletions that prevent its response to amlodipine. These negative results also rule out a detectable fluorescence-ratio contribution from amlodipine accumulated intracellularly.
2. Crucially, we provided electrophysiological evidence that amlodipine activates both native CRAC currents in HEK293 cells with endogenous levels of STIM and Orai and larger CRAC currents in cells co-expressing STIM1 and Orai1, with the expected biophysical properties (Figure 2C-H).^4^ The amlodipine-evoked currents were of similar size to the currents elicited in the same HEK293 cells by a standard store-depletion protocol with BAPTA in the recording pipette, about 0.2-0.5 pA/pF for native CRAC current and 20-50 pA/pF when STIM1 and Orai1 are overexpressed.
3. In this study (Figure 1F and G),^4^ and in the earlier study where we originally identified Ca_v_1.2 channel blockers as activators of Ca^2+^ signaling in glioblastoma cells using a small-molecule screen of a library of 1650 compounds (Figure S2A),^6^ we showed that amlodipine at 0.5-20 μm significantly and dose-dependently elicited a Ca^2+^ signal when the measurement was made with fluo-4, a dye similar to Cal520 and for which overlap with the amlodipine fluorescence spectrum is not an issue.
4. Unlike amlodipine, the drugs diltiazem, nifedipine, verapamil, mibefradil, and BayK8644 do not fluoresce at the wavelengths monitored for fura2 spectra. These drugs activated a similar Ca^2+^ entry (Figure 2M-R; Figure S2; Figure S3A-D).^4^
5. The fura-2 individual-wavelength intensity plots in Bird et al. (Figures 1 and 2)^5^ are irrelevant, because the relevant measurement is the fluorescence ratio F_340_/F_380_. The fluorescence ratio F_340_/F_380_ for amlodipine by itself is slightly less than 1. Hence, to the extent that uptake of amlodipine made any contribution to the ratiometric signal, uncorrected amlodipine fluorescence would depress the true fura-2 ratiometric signal and underestimate the actual increase in cytoplasmic Ca^2+^, as we have documented previously (Figure S2J and K).^6^
6. In Figures 3, 4C, and 4D, Bird et al.^5^ showed that 20 µm amlodipine and diltiazem activate a significant Ca^2+^ signal, though they dismissed it as small or transient.^5^ Contrary to what was implied by Bird et al., we did not claim that Ca_v_1.2 channel blockers activate SOCE to the same extent as maximal activation by thapsigargin; maximal SOCE is not a pre-requisite for physiological significance.
7. SiRNA knockdown (Figure 2I and J)^6^ and CRISPR/Cas9 knockout of STIM1/2 and Orai1/2/3 and cDNA rescue (Figure 2K-R; Figure S2A-L)^4^ showed that members of all major classes of Ca_v_1.2 channel blockers, namely dihydropyridines (amlodipine, nifedipine), verapamil (a phenylalkylamine), and diltiazem (a benzothiazepine) activate Ca^2+^ entry but only when at least one STIM isoform and one Orai isoform are expressed.
8. Diltiazem triggered STIM1 and Orai1 puncta formation and colocalization, induced Förster resonance energy transfer (FRET) between STIM1-eYFP and CFP-Orai1 (Figure 3A-I; Figure 5A-C) and altered STIM1 intramolecular FRET (Figure 5D-H).^4^
9. Bird et al. suggested that amlodipine depletes the endoplasmic reticulum (ER) stores based on measurements with the cytosolic Ca^2+^ indicator Cal520 but this seemed to become evident only with amlodipine at 40 and 100 µm,^5^ a concentration range higher than what we used. We directly measured Ca^2+^ stores using a genetically encoded ER-targeted indicator and showed that 20 µm amlodipine and as high as 50 µm diltiazem failed to alter ER Ca^2+^ content, whereas the subsequent addition of 1 µm ionomycin caused the same extent and rate of ER store depletion in Ca_v_1.2 channel blockers-treated and vehicle-treated cells (Figure 4C-I).^4^ At 20 μm amlodipine, Bird et al. found no Ca^2+^ release from stores in Figures 4A-D, 5A-C, and 5D-E; and very minimal apparent release from stores even in the presence of 1 m m Gd^3+^ to block extrusion of Ca^2+^ by pumps (Figure 5F-G).^5^ Thus, Bird et al. arrived at exactly the conclusion reached in our study: Amlodipine (20 μm) elicits Ca^2+^ influx without depleting stores. A conservative statement of the difference between the two papers on this point is that the amlodipine-evoked Ca^2+^ signal is quantitatively less in Bird et al. under their conditions and with the Ca^2+^ dye used, Cal520.
10. STIM1 dimerization assays on ER membranes isolated from amlodipine- and diltiazem-treated HeLa cells showed that, by comparison to membranes from vehicle-treated cells, amlodipine and diltiazem induced a significantly higher STIM1 dimerization at Ca^2+^ concentrations ranging from 300 μm to 1 m m, demonstrating that treating cells with Ca_v_1.2 channel blockers renders STIM1 prone to activation at resting ER Ca^2+^ concentrations (Figure 5I and J).^4^
11. *Xenopus* STIM1 is activated by store depletion while being insensitive to activation by amlodipine, and a truncated human STIM1 mimicking *Xenopus* STIM1 and lacking an N-terminal region (residues 31-40) loses activation by amlodipine but retains activation by store depletion (Figure 6A-L),^4^ arguing that Ca_v_1.2 channel blockers act, directly or indirectly, on the ER luminal N-terminal domain of STIM1. We also presented evidence that amlodipine and diltiazem require an intermediate step or pathway to activate STIM (Figure 6M and N).^4^

## The Lines of Evidence Above Were Not Acknowledged by Bird et al.

The pharmacokinetics and pharmacodynamics properties of drugs are heavily influenced by binding to plasma proteins, which affect drug distribution, absorption, and half-life.^7^ Drug concentrations in plasma of patients poorly correlate with clinical effects and can serve in monitoring only when protein binding is relatively low (less than 50%).^8^ Concentrations of Ca_v_1.2 channel blockers measured in the plasma of treated patients distribute over a wide range of ∼1-1 μm and 80%-99% of these drugs are in a protein-bound state.^9^ The high half-life and volume of distribution of amlodipine indicates a high absorption and distribution into tissues.^9^ While 0.5 μm amlodipine might be higher than the quoted range in serum of patients, it is not clear how either the concentration applied in our short-term in vitro experiments, or the plasma concentrations measured in patients during long-term treatment relate to amlodipine concentration at its apparent site of action on STIM in the ER lumen of arterial myocytes. Even if we could be sure that the ER concentrations of amlodipine in arterial myocytes of patients were lower than in our in vitro experiments, given that we showed that the cellular action of amlodipine and diltiazem on STIM is indirect, *via* an unknown intermediate step or steps, it would be premature to rule out a cumulative effect of prolonged ER exposure to low concentrations of these drugs. As noted by Bird et al. (Figure S2),^5^ amlodipine accumulates into intracellular compartments including the ER, its site of action on STIM. Therefore, our data are compelling. The Ca^2+^ channel blockers-induced STIM-Orai sensitization exists, and could limit the blockers’ therapeutic effectiveness. We also acknowledge that the drugs concentration at which this pathway is robustly activated is much higher than the clinically therapeutic plasma concentrations.

Bird et al. provided “real-world” meta-analysis comparing incidence of heart failure between placebo patients and patients with no prior history of cardiovascular disease and newly prescribed a monotherapy of either Ca_v_1.2 channel blockers or other anti-hypertensive medications. They assessed the risk of developing heart failure in the following year of follow-up after a minimum of 6 months of drug intake.^5^ This analysis of newly hypertensive patients, where Ca_v_1.2 expression in myocytes would be high and CRAC channel activity would be absent, shows that Ca_v_1.2 channel blockers are effective in these patients, as expected. However, this situation is not a reflection of advanced vascular remodeling, a progressive process that develops over years to decades where Ca_v_1.2 is downregulated, and STIM1/Orai1 increased. Our cross-sectional analysis (Figure 7T)^4^ and that of Bird et al. (Figure 6)^5^ agree that angiotensin converting enzyme inhibitors, beta blockers, angiotensin receptor blockers, and diuretics are more protective against heart failure than Ca_v_1.2 channel blockers. Therefore, Bird et al. manufacture an argument they can win, by blurring the distinction between Ca_v_1.2 channel blockers being effective against hypertension but with possible liabilities—our position—and these blockers being simply ineffective. Contrary to the assertion of Bird et al., we did not claim that Ca_v_1.2 channel blockers are not protective, nor did we suggest they be removed as first line anti-hypertensive drugs.

The study by Bird et al. was accompanied by a commentary and an editorial.^10,11^ Rajagopal and Rosenberg^10^ commented: “One of the major differences from the present study and Johnson et al. was the choice of Ca^2+^-sensitive fluorescent dyes. . .. [Bird et al.] found that amlodipine accumulated within the cytoplasm over several minutes of exposure, dominating the fluorescence signal in fura-2–loaded cells, and thereby mimicking a Ca^2+^ transient. . . Thanks to the rigorous studies in this work, we now have a better understanding of the true effects of amlodipine and CCBs on SOCE, and the clinical utility and safety of amlodipine is no longer in question.” This commentary missed the point that amlodipine fluorescence did not (and, mathematically, could not) register as a spurious increase in the fura2 ratiometric signal, and failed to critically and rigorously discuss and contrast our findings with those of Bird et al.

The editorial by Verkhratsky and Petersen^11^ titled “*How do we clean up the scientific record*” was interesting in that it concluded from the outset that “*the results reported by Johnson et al. are wrong*” and that Bird et al. showed that “*[t]he finding of Johnson et al. is . . . an artifact*,” thus failing to grasp the core experimental findings of our paper and to evaluate critically the claims of Bird et al. Quite ironically, the authors branded our study as a case of “*serious scientific error*,” and used it as a launchpad to offer an extended opinion on problems of scientific rigor and fraud and ways to remedy them. We think a good start would be to avoid omissions and misrepresentations by carefully reading the studies one wishes to comment on.

Verkhratsky and Petersen^11^ went on to state: “. . . how to rectify straightforward errors, which (as illustrated by the case discussed in this editorial) carry dangerous implications? The only way is post-publication testing of the main findings and, in the case of error identification, making the academic community informed through publication, which is of course exactly what is happening in the case discussed here.” This and other statements in this editorial raise a more profound question that goes far beyond the current case. Scientific discourse is healthy and welcomed as it advances our understanding of true biological mechanisms and accelerates the application of this knowledge in the clinic. However, an inherent premise is such discourse needs to be based on the scientific evidence and be judged by the scientific community. In this context, is it appropriate for two scientists, however prominent they are, to self-appoint as the arbiters of the scientific record? A question for the community to address.

In summary, we stand by our conclusion that the current data “indicate caution against the use of L-type Ca^2+^ channels blockers in elderly patients or patients with advanced hypertension and/or onset of cardiovascular remodeling, where levels of STIM and ORAI are elevated.”^4^ A note of caution is precisely that: definitive guidelines for the clinical use of antihypertensive medications can only be established by further research. We look forward to meaningful scientific discussions of our data and data coming from new research in this area.

## Data Availability

There are no data in this manuscript.
